# Identification of a novel pentatricopeptide repeat subfamily with a C-terminal domain of bacterial origin acquired via ancient horizontal gene transfer

**DOI:** 10.1186/1756-0500-6-525

**Published:** 2013-12-09

**Authors:** Sam Manna, Christian Barth

**Affiliations:** 1 Department of Microbiology, La Trobe University, Melbourne, VIC 3086, Australia

**Keywords:** Pentatricopeptide repeat proteins, tRNA methyltransferase, PPR-TGM protein, Horizontal gene transfer, CCCH zinc finger

## Abstract

**Background:**

Pentatricopeptide repeat (PPR) proteins are a large family of sequence-specific RNA binding proteins involved in organelle RNA metabolism. Very little is known about the origin and evolution of these proteins, particularly outside of plants. Here, we report the identification of a novel subfamily of PPR proteins not found in plants and explore their evolution.

**Results:**

We identified a novel subfamily of PPR proteins, which all contain a C-terminal tRNA guanine methyltransferase (TGM) domain, suggesting a predicted function not previously associated with PPR proteins. This group of proteins, which we have named the PPR-TGM subfamily, is found in distantly related eukaryotic lineages including cellular slime moulds, entamoebae, algae and diatoms, but appears to be the first PPR subfamily absent from plants. Each PPR-TGM protein identified is predicted to have different subcellular locations, thus we propose that these proteins have roles in tRNA metabolism in all subcellular locations, not just organelles. We demonstrate that the TGM domain is not only similar to bacterial TGM proteins, but that it is most similar to chlamydial TGMs in particular, despite the absence of PPR proteins in bacteria. Based on our data, we postulate that this subfamily of PPR proteins evolved from a TGM-encoding gene of a member of the Chlamydiae, which was obtained via ancient prokaryote-to-eukaryote horizontal gene transfer. Following its acquisition, the N-terminus of the encoded TGM protein must have been extended to include PPR motifs, possibly to confer additional functions to the protein, giving rise to the PPR-TGM subfamily.

**Conclusions:**

The identification of a unique PPR subfamily which originated from the Chlamydiae group of bacteria offers novel insight into the origin and evolution of PPR proteins not previously considered. It also provides further understanding into their roles in non-organellar RNA metabolism.

## Background

Mitochondria are organelles responsible for providing eukaryotic cells with the energy required to power cellular functions. These rather complex organelles have evolved from an α-proteobacterial endosymbiont, and thus have several features in common with their bacterial ancestors. While mitochondrial genomes can vary significantly in size, they typically only encode proteins involved in ATP synthesis or mitochondrial translation [1,2]. Most proteins involved in mitochondrial and even chloroplast function are therefore nucleus-encoded. Some of these nucleus-encoded gene products have always had nuclear origins, while others were transferred to the nucleus from the mitochondrial genome [3].

Other nucleus-encoded mitochondrial or plastid proteins have been acquired by other means. One of these is the contribution of genes from bacteria that were not involved in the endosymbiotic events that gave rise to these organelles. This event, referred to as ancient or prokaryote-to-eukaryote horizontal gene transfer (HGT) is believed to have been an important driving force in the diversification of life [4,5]. Evidence for this form of HGT has been identified in several eukaryotic genomes, which possess multiple bacterial genes, many of which encode gene products with mitochondrial or plastid functions [4,6-9].

Pentatricopeptide repeat (PPR) proteins are a group of highly diverse nucleus-encoded RNA binding proteins, defined by a tract of repeated 35 amino acid motifs. They are involved in the regulation of multiple aspects of mitochondrial and plastid gene expression, including RNA editing, processing, splicing, stability and translation [10,11]. Despite the fact that most PPR proteins function in these bacterial-derived organelles, prokaryotes do not typically possess PPR proteins. With the exception of studies exploring the expansion of these proteins in plants, the origin and evolution of PPR proteins is not well understood [12-14]. Some PPR-encoding genes have previously been proposed to have been transferred via HGT, including eukaryote-to-eukaryote HGT, and one possible eukaryote-to-prokaryote event, but none has been postulated to have been acquired via a prokaryote-to-eukaryote HGT event [11,15-17]. Several subfamilies of PPR proteins exist, which are classified based on the types of additional domains they contain. One of these subfamilies is the PPR-SMR subfamily, which in addition to their PPR motifs, contain a bacterial-type SMR (small MutS-related) domain [18,19]. Additionally, a recent investigation into PPR proteins in the model protist *Dictyostelium discoideum* and other closely related species, led to the identification of PPR-containing proteins with C-terminal tRNA guanine N-7 methyltransferase (TGM) domains that are similar to bacterial TGM proteins [20]. However, the potential bacterial origins of the SMR or TGM domain-containing PPR proteins have not been explored.

Here, we investigate the evolutionary origins of the PPR-containing tRNA guanine methyltransferases from *D. discoideum* and other closely related protozoa. This led to the identification of a group of proteins with similar features in distantly related eukaryotic lineages, which we have named the pentatricopeptide repeat-containing tRNA guanine methyltransferase (PPR-TGM) subfamily. All members of this subfamily are predicted to have important roles in tRNA metabolism. While the vast majority of PPR proteins have been found and characterised in plant organelles, the PPR-TGM subfamily appears to be absent from plants, making it the first group of PPR proteins not found in plants. We provide evidence that the TGM domains of these proteins not only closely resemble bacterial TGM domains, but that they are most similar to chlamydial TGM domains in particular. Our data support the notion that the high level of similarity between PPR-TGM proteins and chlamydial TGMs is due to an ancient prokaryote-to-eukaryote HGT event, in which a chlamydial TGM-encoding gene was transferred to eukaryotes. Following its eukaryotic acquisition, this chlamydial TGM evolved via the incorporation of PPR motifs to allow the protein to mediate other functions in tRNA metabolism, giving rise to the PPR-TGM subfamily.

## Results and discussion

### Identification of members of the PPR-TGM subfamily

**Figure 1:**
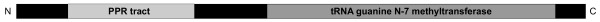
**Conserved domain architecture of PPR-TGM proteins.** Each protein has a PPR tract consisting of 3–7 PPR motifs and a C-terminal tRNA guanine N-7 methyltransferase domain. Additionally, some PPR-TGM proteins contain N-terminal signal peptides (not shown).

Recently, we identified and characterised PtcE, a PPR protein with a bacterial-like tRNA guanine N-7 methyltransferase (TGM) domain in the cellular slime mould *D. discoideum* and other closely related organisms [20]*.* At the time, we believed this PPR protein was only present in the cellular slime mould lineage. In the present study, we investigated the origin and evolution of PtcE, particularly with regard to the potential bacterial origin of the TGM domain. This unexpectedly led to the identification of additional PPR-containing proteins with TGM domains in other eukaryotes. We have named this group of proteins the PPR-TGM subfamily, as all members of this subfamily contain PPR motifs and a C-terminal TGM domain (Figure 1). Based on *in silico* analysis, it is predicted these proteins have roles in the methylation of guanine residues in tRNAs at position 46 to form 7-methylguanosine (m^7^G). This type of methylation makes G46 positively charged and, as it has been shown in yeast, can influence interactions and hydrogen bonding of this nucleotide with C13 and G22 in tRNAs at the tertiary level [21-23]. TGM domains have never been seen in PPR proteins previously and consistent with this, methylation is a function that has not been associated with PPR proteins. Thus, it seems the PPR-TGM subfamily is likely to be a unique group of PPR proteins, with prominent and novel roles in tRNA nucleotide modification.

A total of 22 PPR-TGM proteins were identified, all of which are found in several distantly related eukaryotic lineages including cellular slime moulds, entamoebae, algae and diatoms, but they appear to be absent in plants (Table 1). The identified proteins range in size from 406–1884 amino acids, and each PPR-TGM protein contains a conserved range of approximately 3–7 PPR motifs, as determined using the PPR bioinformatic predictive tool TPRpred [24].

**Table 1:** Complete list of identified PPR-TGM proteins

**Organism**	**NCBI protein accession**	**Probability of PPR (%)**^**1**^	**Number of PPR motifs**^**1**^	**Probability of mitochondrial targeting (%)**^**2**^
**Cellular slime moulds**				
*Dictyostelium discoideum*	XP_646896	100	5	53
*Dictyostelium purpureum*	XP_003288663	100	5	69
*Polysphondylium pallidum*	EFA82229	100	3	71
*Dictyostelium fasciculatum*	EGG13534	97.13	3	15
**Entamoebae**				
*Entamoeba nuttalli*	EKE39146	99.78	3	N/A
*Entamoeba histolytica*	XP_001913841	99.78	3	N/A
*Entamoeba invadens*	XP_004259532	100	5	N/A
**Perkinsozoan protists**				
*Perkinsus marinus*	XP_002764510	100	4	0
**Chlorophyte algae**				
*Ostreococcus lucimarinus*	XP_001417638^3^	100	6	9
*Ostreococcus tauri*	XP_003079103^3^	100	6	82
*Ostreococcus tauri*	XP_003079628	100	6	8
*Bathycoccus prasinos*	CCO19295^3^	99.76	3	2
*Bathycoccus prasinos*	CCO16496	100	6	2
*Micromonas pusilla*	XP_003056532^3^	100	5	75
*Micromonas pusilla*	XP_003059060	100	5	92
*Micromonas* sp.	XP_002500331	100	6	2
**Cryptophyte algae**				
*Guillardia theta*	EKX33941	100	4	92
*Guillardia theta*	EKX51242	100	4	13
*Guillardia theta*	EKX45176	100	6	43
**Haptophyte algae**				
*Emiliania huxleyi*	EOD30717	100	7	22
**Diatoms**				
*Thalassiosira oceanica*	EJK76724	99.96	4	1
*Phaeodactylum tricornutum*	XP_002177626	100	3	23

^1^:Probability scores for the presence of PPR motifs were determined using TPRpred.

^2^:Probability scores for analysis of mitochondrial targeting were determined using Mitoprot.

^3^:Sequences contain a CCCH-type zinc finger motif.

### Different PPR-TGM proteins are predicted to have different subcellular localisations

Interestingly, despite the notion that most PPR proteins are either mitochondrially or plastid targeted, only a few of the newly identified PPR-TGM proteins are predicted to have N-terminal mitochondrial targeting signals according to the predictive software program Mitoprot [25] (Table 1). Similarly, the algal and diatomic PPR-TGM proteins demonstrate very low probabilities of chloroplast targeting (data not shown). One exception to this was a PPR-TGM protein from the alga *Guillardia theta* [NCBI protein accession no. EKX33941], which was not only predicted to contain a mitochondrial targeting signal (Table 1), but was also predicted to contain a chloroplast targeting signal (TargetP probability score: 83%) [26]. The plastid and mitochondrial targeting prediction was confirmed using several targeting software tools (data not shown). Thus, it is possible that this PPR-TGM protein may localise to both organelles.

The apparent lack of organelle targeting for most of the other PPR-TGM proteins could be due to the inability of the predictive software programs to detect non-conventional targeting signals in these proteins. This is because these software programs are designed to detect traditional signal peptides located at the N-terminus of the protein and not internal or C-terminal targeting signals [25,26]. Alternatively, the absence of organelle targeting signals can be explained by the role some of these PPR-TGM proteins are predicted to have in cytoplasmic tRNA metabolism. Unexpectedly, PPR-TGM proteins were also identified in members of the *Entamoeba* genus, and to our knowledge, this is the first report of PPR proteins in this genus. The entamoebae are a group of protists, which instead of mitochondria, possess mitosomes, degenerate mitochondrial-like organelles [27,28]. However, mitosomes do not possess their own DNA [29,30] and thus, there is no obvious requirement for PPR proteins in mitosomes. The presence of PPR-TGM proteins in *Entamoeba* species therefore supports the hypothesis that the PPR-TGM proteins that lack obvious mitochondrial or plastid targeting signals may indeed be involved in cytoplasmic tRNA metabolism. This suggests that PPR proteins play a significantly greater role in cytoplasmic RNA processing than originally expected, and may provide insight into the early stages of the evolution of the PPR motif.

In most of the organisms in which PPR-TGM proteins are found, only a single PPR-TGM-encoding gene was identified. However, this was not the case in algae, where most genomes were found to encode at least two PPR-TGM proteins (Table 1). This raises the question as to why algae would require multiple PPR-TGM proteins, while other eukaryotes such as diatoms and cellular slime moulds only require one. The alga *Ostreococcus tauri* has two PPR-TGM proteins, one with a mitochondrial targeting signal and one without (Table 1), suggesting that the PPR-TGM proteins in this alga may mediate tRNA metabolism in different subcellular locations, including the cytoplasm, mitochondria, and possibly chloroplasts, as is the case in *G. theta*. However, it is noteworthy that the two PPR-TGM proteins found in *Bathycoccus prasinos* are both predicted to be cytoplasmic, while *Micromonas pusilla* has two mitochondrial PPR-TGM proteins (Table 1). The PPR-TGM proteins from these algae demonstrate that alternate subcellular localisation does not always explain the presence of multiple PPR-TGM proteins in the same organism, and that there are therefore likely to be other reasons which remain to be elucidated.

### Evidence of a gene duplication and subsequent sequence divergence in the chlorophyte algae lineage

**Figure 2:**
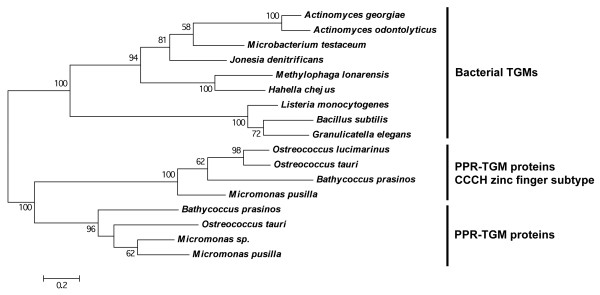
**Phylogenetic tree displaying the evolutionary relationship of chlorophyte algal PPR-TGM proteins.** Amino acid sequences were aligned using MUSCLE using bacterial TGMs as the outgroup. The maximum likelihood phylogeny tree was generated using the Jones-Taylor-Thornton model with the software program MEGA5. The scale represents the number of substitutions per site. Statistical support for the branches was ascertained via bootstrapping (100 replicates). Only bootstrap values greater than 50% are shown.

In addition to the PPR tract and TGM domain, one of the PPR-TGM proteins from chlorophyte algae *Ostreococcus lucimarinus, O. tauri, B. prasinos* and *M. pusilla* [NCBI protein accession no. XP_001417638, XP_003079103, CCO19295 and XP_003056532, respectively] also contain a CCCH-type zinc finger motif. Phylogenetic analysis of these proteins revealed that the PPR-TGM subgroup containing the CCCH-type zinc finger motifs have diverged significantly in sequence as compared to the traditional PPR-TGM proteins found in the same species (Figure 2). Given that most chlorophyte algae possess one PPR-TGM and one PPR-TGM CCCH-type zinc finger protein, it is likely that the CCCH-type zinc finger subgroup arose from a gene duplication event of the traditional PPR-TGM encoding gene in this lineage, and following sequence divergence, evolved this additional motif. CCCH-type zinc finger proteins are known for their affinity to RNA and similarly to PPR proteins, they mediate several functions in RNA biogenesis [31-34]. The zinc finger motifs in algal PPR-TGM proteins are therefore likely to facilitate the function of the PPR motifs and contribute to RNA binding and metabolism. While the requirement for a second RNA recognition motif in addition to the RNA-binding capabilities of the PPR motif is not clear, the fact that some PPR-TGM proteins do not have the CCCH-type zinc finger, while others from the same species of alga do, implies the two proteins may have different functions. Also noteworthy was the presence of only one PPR-TGM protein in the chlorophyte alga *O. lucimarinus,* which was of the CCCH-type zinc finger subtype. This possible gene loss of the traditional PPR-TGM protein may indicate functional redundancy, or a function performed by this protein that is no longer required in this organism.

### PPR-TGM proteins display strong sequence similarity to chlamydial tRNA guanine methyltransferases

Similarly to the small MutS-related (SMR) domain in PPR-SMR proteins, we previously found that the TGM domain in the PPR-TGM protein PtcE shared sequence similarity to bacterial TGMs [20]. Our current analyses not only confirmed the high level of sequence similarity between all PPR-TGM proteins and bacterial TGMs, but more specifically showed that most of the bacterial TGMs were from members of the Chlamydiae phylum, including the genera *Chlamydia, Chlamydophila, Waddlia, Simkania* and *Candidatus Protochlamydia*. This was supported by a series of amino acid alignments, which further demonstrated a higher level of sequence similarity to chlamydial TGMs rather than to bacterial TGMs in general (Additional files 1 and 2). Also evident was the presence of an N-terminal extension of ~300-500 amino acids in the PPR-TGM proteins, which is absent from the chlamydial and other bacterial TGMs. The additional N-terminal sequences correspond to the location of the PPR tract, indicating a lack of PPR motifs in the chlamydial and other bacterial TGMs. This was confirmed via the inability to detect any PPR motifs in chlamydial and other bacterial TGMs using TPRpred analysis (data not shown), and is consistent with the PPR motif being an exclusively eukaryotic motif. The fact that chlamydial TGMs still display significant levels of sequence similarity to PPR-TGMs proteins despite the presence of the significantly sized N-terminal extension, further attests to the high level of sequence similarity of this subfamily to TGMs from the Chlamydiae.

### The PPR-TGM subfamily evolved from a chlamydial tRNA guanine methyltransferase inherited via ancient horizontal gene transfer

**Figure 3:**
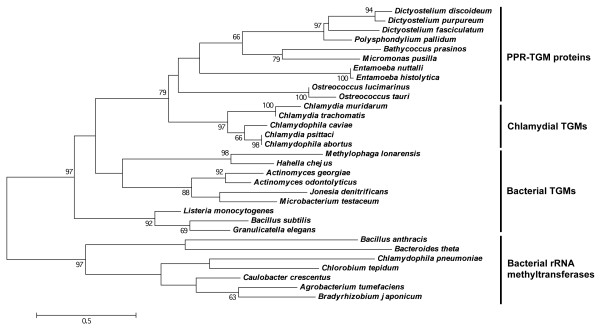
**Phylogenetic tree displaying the relationship of chlamydial TGMs to the TGM domain of PPR-TGM proteins.** Amino acid sequences were aligned using MUSCLE. Bacterial rRNA methyltransferases were used as the outgroup. The maximum likelihood phylogeny tree was generated using the Jones-Taylor-Thornton model with the software program MEGA5. The scale represents the number of substitutions per site. Statistical support for the branches was ascertained via bootstrapping (100 replicates). Only bootstrap values greater than 50% are shown.

**Figure 4:**
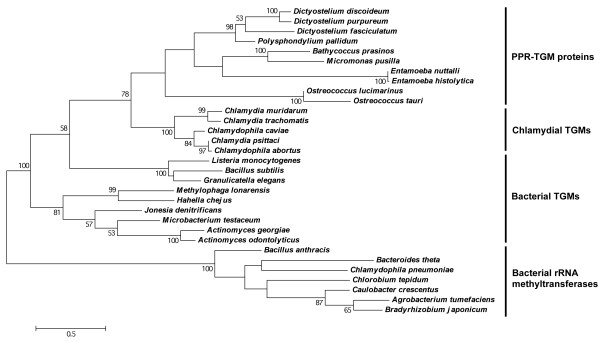
**Phylogenetic tree displaying the relationship of chlamydial TGMs to full length PPR-TGM proteins.** Amino acid sequences were aligned using MUSCLE. Bacterial rRNA methyltransferases were used as the outgroup. The maximum likelihood phylogeny tree was generated using the Jones-Taylor-Thornton model with the software program MEGA5. The scale represents the number of substitutions per site. Statistical support for the branches was ascertained via bootstrapping (100 replicates). Only bootstrap values greater than 50% are shown.

Given the non-α-proteobacterial Chlamydiae phylum is not considered of having played a role in the evolution of endosymbiotic-derived organelles, the higher level of sequence similarity between PPR-TGM proteins and chlamydial TGMs compared to other bacterial TGMs was rather perplexing. To find an explanation for the unexpected sequence similarity, the evolution of the PPR-TGM subfamily was investigated using phylogenetic reconstruction. The phylogenetic tree containing the TGM domain from PPR-TGM proteins, as well as chlamydial and other bacterial TGMs, was found to be incongruent with the universal tree of life (Figure 3). In particular, the chlamydial TGMs formed a sister group with the PPR-TGM proteins (bootstrap value: 79%, Figure 3), and the former displayed less similarity to the other bacterial TGMs. The statistical support between these sister groups of proteins was even supported when the full length PPR-TGM amino acid sequences were used in the phylogenetic analysis (bootstrap value: 78%, Figure 4), despite the presence of the large PPR-containing N-terminal extension. A similar tree topology was also observed using Phylogeny.fr [35], a second phylogenetic analysis program providing further support for the PPR-TGM/chlamydial TGM sister relationship (bootstrap value: 98%, Additional file 3). The observed tree incongruence of these trees (Figures 3 and 4, and Additional file 3) with the universal tree of life is characteristic of an ancient horizontal gene transfer (HGT) event. Thus, it appears that the PPR-TGM subfamily originated from a single TGM-encoding gene obtained from an ancient chlamydial species via HGT by a eukaryotic recipient. Following its transfer, the N-terminus of the encoded protein was extended and PPR motifs were incorporated, giving rise to the PPR-TGM subfamily. This phenomenon of gene transfer has been reported extensively, and has been found to occur between prokaryotic and eukaryotic lineages in both directions [3,7-9,36].

There are several pieces of evidence that support the notion of the origin and evolution of the PPR-TGM subfamily from a chlamydial TGM-encoding gene acquired via HGT. One of the major hallmarks for an ancient prokaryote-to-eukaryote HGT event is the punctate distribution of a bacterial gene in eukaryotic lineages [4,36]. This was observed in the current study with the distribution of bacterial-like TGM proteins in evolutionary distinct eukaryotic lineages, including algae, diatoms, entamoebae and cellular slime moulds. In addition to this, all of these lineages have previously been reported to possess genes acquired via HGT from prokaryotes [5,6,36-38].

The second indicator for ancient HGT is tree incongruence from the expected phylogenetic distribution of the selected organisms [4,36]. This was demonstrated by the chlamydial TGM proteins forming sister groups with PPR-TGM proteins rather than other bacterial TGM proteins (Figures 3 and 4, and Additional file 3). This observation is also supported by the fact that algal, diatomic and cellular slime mould genomes in particular have also been found to encode genes predicted to be specifically of chlamydial origin in previous studies [6,38], which is consistent with the higher level of similarity of PPR-TGM proteins to chlamydial TGMs. While HGT involving a chlamydial donor has not been reported in the *Entamoeba* genus, this form of gene transfer has been predicted to occur in this lineage from other bacterial donors [37,39,40].

Ancient HGT has been reported extensively in amoebae. Free-living amoebae are in constant interaction with bacteria in the environment, as they rely on them as a food source, they can act as hosts for intracellular pathogenic bacteria, and they often form symbioses with intracellular bacteria, including associations with several chlamydial species [41,42]. Thus, free-living amoebae are constantly exposed to foreign DNA, providing a natural opportunity for gene transfer events to take place [41]. Such protists in which this event has been reported, not only include *Acanthamoeba castellanii, Entamoeba histolytica,* and *Hartmannella vermiformis,* but also *D. discoideum*[5,37]*.*

Additionally, prokaryote-to-eukaryote HGT was found to be a common occurrence in plants. In particular, Moustafa et al. [6] identified over 50 plant genes predicted to be of chlamydial origin with most having functions in chloroplasts. Another study by Becker et al. [38] provided evidence for the transfer of chlamydial genes to plant genomes by identifying over 30 chlamydial genes in plant genomes predicted to have been acquired via HGT, including multiple RNA methyltransferases [38]. Several of these chlamydial genes were also found in diatoms, algae and even cellular slime moulds [38], including many of the genera in which we have identified PPR-TGM proteins.

Becker et al. [38] found three chlamydial genes in the *D. discoideum* genome, one of these encodes a queuine tRNA-ribosyltransferase*.* The *D. discoideum* queuine tRNA-ribosyltransferase is predicted to have a mitochondrial targeting signal (Mitoprot probability score: 81%), and also seems to have homologs in most of the eukaryotic lineages which also possess PPR-TGM proteins, including algae and diatoms (data not shown). Thus, given the evidence for chlamydial HGT in several eukaryotic lineages, including the acquisition of a gene encoding a mitochondrially targeted tRNA nucleotide modification enzyme, it is not unreasonable to postulate that the PPR-TGM proteins with similar features have evolved from a chlamydial TGM-encoding gene acquired via HGT. However, whether this event occurred once in the common ancestor of all PPR-TGM-containing eukaryotic lineages, and was subsequently lost in others, or if the event occurred in one lineage (for example, the cellular slime mould lineage), followed by a series of eukaryote-to-eukaryote HGT events, remains to be determined. In any case, the consistently higher levels of sequence similarity and HGT origins of the PPR-TGM subfamily with chlamydial TGMs demonstrates a common ancestry of the members of this family, a rather unique characteristic for a PPR subfamily in distantly related eukaryotic lineages.

## Conclusions

Given that several of the characterised tRNA methyltransferases do not have PPR motifs, it is clear that PPR motifs are typically not required for methylation. Thus, it seems that during evolution, the N-terminus of the PPR-TGM proteins was extended to incorporate PPR motifs, which would confer a function in addition to methylation, possibly in other aspects of tRNA metabolism. Alternatively, it is possible that PPR-TGM proteins are a product of a gene fusion event between the recently acquired chlamydial TGM-encoding gene, and a pre-existing PPR-encoding gene in the recipient genome. Nonetheless, the notion of the PPR-TGM subfamily having originated from an existing bacterial gene acquired by HGT not only sheds light on the evolution of a novel PPR subfamily outside of plants, but also presents a novel mechanism for the evolution of PPR proteins containing additional domains, such as the PPR-SMR proteins, which may have not been considered previously. Moreover, the identification of the first PPR proteins in the amitochondrial *Entamoeba* genus provides invaluable information required to help unravel evolutionary complexities such as the origin of the PPR motif, and why this motif is essential for the regulation of gene expression in organelles, but is absent in bacterial ancestors.

## Methods

### Sequence analysis

All PPR-TGM proteins were originally identified using the PPR-TGM protein, PtcE, as a query sequence in the NCBI protein database (BLASTP). The predicted PPR-TGM proteins were confirmed to be genuine members of this subfamily using NCBI BLASTP and InterProScan [43], which uses several protein signature and motif recognition software programs. TPRpred [24] was also used as a more sensitive tool to determine the number and associated probabilities of PPR motifs. For comparison of PPR-TGM proteins with bacterial proteins, the chlamydial and other bacterial TGMs with the highest similarity to PPR-TGM proteins were selected. Amino acid alignments were performed with CLUSTAL W [44] using the standard parameters.

### Phylogenetic analysis

Phylogenetic analyses were performed using the software package MEGA 5 [45]. The amino acid sequences of either algal PPR-TGM proteins, or the selected PPR-TGM proteins, chlamydial TGMs and bacterial TGMs were aligned using MUSCLE [46]. As appropriate, either bacterial rRNA methyltransferase sequences or bacterial TGMs were used as the outgroup. The maximum likelihood trees were generated using the Jones-Taylor-Thornton substitution model [47] and alignment gaps were removed. The nearest-neighbour-interchange heuristic method was employed. The maximum parsimony method was used if less than 100 sites were in common. The BIONJ method was used for common sites greater than 100 in conjunction with the maximum composite likelihood pairwise distance matrix. Statistical support for the branches was ascertained via bootstrapping (100 replicates). The topologies of the trees were confirmed with a second program, Phylogeny.fr [35] using the same parameters.

## Abbreviations

PPR: Pentatricopeptide repeat; TGM: tRNA guanine N-7 methyltransferase; PPR-TGM: Pentatricopeptide repeat-containing tRNA guanine N-7 methyltransferase; HGT: Horizontal gene transfer; SMR: Small MutS-related.

## Competing interests

The authors declare that they have no competing interests.

## Authors’ contributions

SM devised the study and performed all bioinformatic and evolutionary analyses, in addition to writing and drafting the manuscript. CB also devised the study and contributed to the writing and drafting the manuscript. All authors read and approved the final manuscript.

## Authors’ information

SM was the recipient of an Australian Postgraduate Award.

## Supplementary Material

Additional file 1 **Amino acid sequence alignment of PPR-TGM proteins with bacterial TGMs.** Bacterial TGM sequences used in the alignment include those from *Jonesia denitrificans* [Jd, accession no. YP_003160858], *Microbacterium testaceum* [Mt, accession no. YP_004223305], *Actinomyces georgiae* [Ag, accession no. ZP_16358116], *Nitratiruptor* sp. [Ns, YP_001356223] and *Sulfuricurvum kujiense* [Sk, YP_004060596]. PPR-TGM sequences used in the alignment include those from *Dictyostelium discoideum* [Dd, accession no. XP_646896], *Bathycoccus prasinos* [Bp, accession no. CCO16496], *Entamoeba histolytica* [Eh, accession no. XP_001913841] and *Ostreococcus tauri* [Ot, accession no. XP_003079103]. Identical (*), conserved (:) and semi-conserved (.) amino acids are indicated. The PPR-containing region of the PPR-TGM proteins is denoted by the red box and the TGM domain of all sequences is denoted by the green box. Click here for file

Additional file 2 **Amino acid sequence alignment of PPR-TGM proteins with chlamydial TGMs.** Chlamydial TGM sequences used in the alignment include those from *Parachlamydia acanthamoebae* [Pa, accession no. ZP_06300024], *Candidatus Protochlamydia amoebophila* [Cpa, accession no. YP_008284], *Waddlia chondrophila* [Wc, accession no. YP_003709095], *Simkania negevensis* [Sn, YP_004671640], *Chlamydophila pneumoniae* [Cp, YP_005662431] and *Chlamydia psittaci* [Cps, accession no. AFS24598]. PPR-TGM sequences used in the alignment include those from *Dictyostelium discoideum* [Dd, accession no. XP_646896], *Bathycoccus prasinos* [Bp, accession no. CCO16496], *Entamoeba histolytica* [Eh, accession no. XP_001913841] and *Ostreococcus tauri* [Ot, accession no. XP_003079103]. Identical (*), conserved (:) and semi-conserved (.) amino acids are indicated. The PPR-containing region of the PPR-TGM proteins is denoted by the red box and the TGM domain of all sequences is denoted by the green box. Click here for file

Additional file 3 **Phylogenetic tree displaying the relationship of PPR-TGM proteins to chlamydial TGMs using phylogenetic software program, Phylogeny.fr.** Amino acid sequences were aligned using MUSCLE. Bacterial rRNA methyltransferases were used as the outgroup. The maximum likelihood phylogeny tree was generated using the Jones-Taylor-Thornton model. The scale represents the number of substitutions per site. Statistical support for the branches was ascertained via bootstrapping (100 replicates). Click here for file
