# Supplementary material for: Identification of a novel pentatricopeptide repeat subfamily with a C-terminal domain of bacterial origin acquired via ancient horizontal gene transfer
Source: BMC Res Notes. 2013 Dec 9;6:525. doi: 10.1186/1756-0500-6-525 (PMC4029402; doi:10.1186/1756-0500-6-525)
Supplement: Additional file 1 — Amino acid sequence alignment of PPR-TGM proteins with bacterial TGMs. Bacterial TGM sequences used in the alignment include those from Jonesia denitrificans [Jd, accession no. YP_003160858], Microbacterium testaceum [Mt, accession no. YP_004223305], Actinomyces georgiae [Ag, accession no. ZP_16358116], Nitratiruptor sp. [Ns, YP_001356223] and Sulfuricurvum kujiense [Sk, YP_004060596]. PPR-TGM sequences used in the alignment include those from Dictyostelium discoideum [Dd, accession no. XP_646896], Bathycoccus prasinos [Bp, accession no. CCO16496], Entamoeba histolytica [Eh, accession no. XP_001913841] and Ostreococcus tauri [Ot, accession no. XP_003079103]. Identical (*), conserved (:) and semi-conserved (.) amino acids are indicated. The PPR-containing region of the PPR-TGM proteins is denoted by the red box and the TGM domain of all sequences is denoted by the green box. [file 1756-0500-6-525-S1.pdf]

```
Jd_TGM          ------------------------------------------------------------
Mt_TGM          ------------------------------------------------------------
Ag_TGM          ------------------------------------------------------------
Ns_TGM          ------------------------------------------------------------
Sk_TGM          ------------------------------------------------------------
Dd_PPR-TGM      MDLKRKFGNSLGSTAPTSIGILGGGGGGVSITKLIVKNIPKSISKNEIIENLKKIIQNPS 60
Bp_PPR-TGM      ------------------------------------------------------------
Eh_PPR-TGM      ------------------------------------------------------------
Ot_PPR-TGM      ------------------------------------------------------------

Jd_TGM          ------------------------------------------------------------
Mt_TGM          ------------------------------------------------------------
Ag_TGM          ------------------------------------------------------------
Ns_TGM          ------------------------------------------------------------
Sk_TGM          ------------------------------------------------------------
Dd_PPR-TGM      INITIPITLPPDLIGKVDADTTISFLSSQNLSQLSIIKNLVNGKTIGDKKVIVDFYDFKK 120
Bp_PPR-TGM      ------------------------------------------------------------
Eh_PPR-TGM      ------------------------------------------------------------
Ot_PPR-TGM      ----------------------------------------------------MAKRKKRP 8

Jd_TGM          ------------------------------------------------------------
Mt_TGM          ------------------------------------------------------------
Ag_TGM          ------------------------------------------------------------
Ns_TGM          ------------------------------------------------------------
Sk_TGM          ------------------------------------------------------------
Dd_PPR-TGM      VIPTPTPIPTPTPPTKTQEESNKKIKLTNEKPKEKKPKEKKPKTTPPPTTTTPNEPTIAT 180
Bp_PPR-TGM      ------------------------MAAKRKNEESREVSDTRENYKKKSSNGVVVGGDFAR 36
Eh_PPR-TGM      -----------------------------------------------MSKESTTPNEINE 13
Ot_PPR-TGM      WLDASVVRNARARDGEKARERRERERAFREKCPERFVEEEKERESERERDARARDGDFKA 68

Jd_TGM          ------------------------------------------------------------
Mt_TGM          ------------------------------------------------------------
Ag_TGM          ------------------------------------------------------------
Ns_TGM          ------------------------------------------------------------
Sk_TGM          ------------------------------------------------------------
Dd_PPR-TGM      ATATTTTTETTETEKTTEINEEKKVNQKKNKKLNDKKEEKEKEKEVKKEEEPKKESIVRN 240
Bp_PPR-TGM      FVAEQQQKDTSKEEHLSSLNELQPTPYSQLAKLDEGMNRCFRANRKRSRDAQTLLKRAKK 96
Eh_PPR-TGM      RKIEEKPHKEEKKMKKQRYKKEEILGNPLEEYFEKVSKNVAPYNPIGRAEEEIEEG---F 70
Ot_PPR-TGM      STSGRGGRRNDREGADGRRKGHKGDGSPDDSFSTDP--RTFPTHATFQTREGLSNDALEW 126

Jd_TGM          ------------------------------------------------------------
Mt_TGM          ------------------------------------------------------------
Ag_TGM          ------------------------------------------------------------
Ns_TGM          ------------------------------------------------------------
Sk_TGM          ------------------------------------------------------------
Dd_PPR-TGM      NRAVINKITSQMSSCAEAKDYVQLVKAYNYLKKIGAKPDHITYGVMLNACVRCQEYKKVK 300
Bp_PPR-TGM      LKEVKFFNRLIRDFGNDKQFGFAEEAFRILEKECNIEPNAYSYTNLLNACVRVGELKRAR 156
Eh_PPR-TGM      SQKSIFYINKLFADAAQERNYGKVIYLYKKLIESGIKEDLHTITNIINSAARVGDGILVD 130
Ot_PPR-TGM      RRSRVEAVTKDIAMCARHKQLRRACRAFVQLIEDGMVPSSYTYASLLNAYVNTGSMDG-A 185

Jd_TGM          ------------------------------------------------------------
Mt_TGM          ------------------------------------------------------------
Ag_TGM          ------------------------------------------------------------
Ns_TGM          ------------------------------------------------------------
Sk_TGM          ------------------------------------------------------------
Dd_PPR-TGM      EVFQDAVKDGNA--NEVVYTIYVKALCEI-DMDESFATIKGMIE-----TKPNIRTFNSI 352
Bp_PPR-TGM      EVFQKMERDCDEKPNEVTCTVFIKGLCEEGLIDEALELVKDMVRGSASRPRANVRTFSTI 216
Eh_PPR-TGM      KTWIR-MKQLGLKANEVTRTVSVKGYFAAGLVEKAMYTYYCMD------NRNNIRSINAA 183
Ot_PPR-TGM      EALMERMSEVGCAPNVVAYTTMLKGYMLVADVDAAWRLLEGMKHP----VAPDIRAVNTY 241
```

```
Jd_TGM          ------------------------------------------------------------
Mt_TGM          ------------------------------------------------------------
Ag_TGM          ------------------------------------------------------------
Ns_TGM          ------------------------------------------------------------
Sk_TGM          ------------------------------------------------------------
Dd_PPR-TGM      FRGCIRSGDIEITKSLIQLM-KANEIYPDST-----TIEYLIKIYSHHLMVQEIWDLLGK 406
Bp_PPR-TGM      LRNCVRYRDVHSAEATFSLMRECFDVLPDAA-----CYEYLSKSYASRLDVEKAEKTLNE 271
Eh_PPR-TGM      IRGLLRLGNSKQ----IHTFTKHPINAEDMT-----TKEYLTALYS-------------- 220
Ot_PPR-TGM      IRVCVRCGSLTXXXXXXXSVKKNEHLRWTLDPSFGHCQFWCAGKCERGANCRFYHDPSIE 301

Jd_TGM          ------------------------------------------------MNN----DAASV 8
Mt_TGM          ------------------------------------------------MSG----RKVPV 8
Ag_TGM          ------------------------------------------------MSAGAPHSRAPG 12
Ns_TGM          -----------------------MPHIVAKHFHHFDTPVTKEGYRFLWFARPLVNAKDEL 37
Sk_TGM          -----------------------MPHLNLESFKPLTYPTQCGEVEFLFQADYKNPDEGAL 37
Dd_PPR-TGM      VYERMQG-----QISPICFSRLSLASLLAGDIKSSVKALGITDDILSKAPRSTQTTHKNK 461
Bp_PPR-TGM      LELQQDENGKVLNIPASALASLAGVAATVGKVDVAKRAIAKCRERADEEQRNAEQFSSNA 331
Eh_PPR-TGM      ---------------------IEQEISLVRKIMESINNEDEMSATSLINLATLNCLIGDV 259
Ot_PPR-TGM      QVDASARETDVNDMLAHLYVNHAHATAMSGDVKQCFKSLAKAAESFAQDDDGNAAGLKDR 361

Jd_TGM          SVSPDEPGDGDAVAPVEEMGEGQVEAGEHRPKIVSFVSRSTRLKGRQQRAWDELAP---- 64
Mt_TGM          RHTP----EGQPNTP-----DARDTAPVFRDKPVSFVRRSGRMSEAQDRAWAELSP---- 55
Ag_TGM          VVGPVPGVPRAPAPGASPRRRPGQEGGVFMARTKSFTRRSRELPPNLRRTWEAVAP---- 68
Ns_TGM          VAVEYDKKPFLLTIKPKRDGTFVIKGDKITRLSPTVLMKMALKNFCDLAQCDVLYD---- 93
Sk_TGM          IATRYKGREFFLVYKN--GAKKLLKSDKITRPSPNFILKHALLAYAECSGSPILDS---- 91
Dd_PPR-TGM      GKLSEKNKISSSLFERINKQEINEESDRVRNYLSKMTESRSKIKYHNMESFNRIYFSKNT 521
Bp_PPR-TGM      NSSSEKDRSNNQKPNDDSTTPSKSVSNFFKARASDALREIVEIEAFLSSDDDVIRQEAKQ 391
Eh_PPR-TGM      ETGDLLLKEFEDISKRETDARKSIR-LFKKHQVAQLRNRRKHIRTFILNTQPILKEQRK- 317
Ot_PPR-TGM      DERAELFRQTSRDELKREMKRIKAFAERLKNGEQKAPNLDEHFARSLIFSSQILQPPERG 421
                                                                     :

Jd_TGM          -----------------------------LFVIAPPRLMSRTSIAPEAVFDPGEVFG-RS 94
Mt_TGM          -----------------------------VYLLPVERAAAATSVKAGTAADLEHVYG-RA 85
Ag_TGM          -----------------------------RYVIEPRRGVGRTTVAEDFALDPVEVFG-RR 98
Ns_TGM          -----------------------------NLSTIKTSHAQKAREFLKEIDFFIEQFP-KN 123
Sk_TGM          -----------------------------NVDNAPENTHLKTHDALKTISYFAENFP-KN 121
Dd_PPR-TGM      FKNNVNRS-------------IDQQCKPVTVATSPDSTLSSFNGLFNSYKKGGSNFN-SN 567
Bp_PPR-TGM      RAEAFG-----------------VDETDDIVFVHKQRELAETPAIEKFWASRYERKGKAF 434
Eh_PPR-TGM      -----------------------------KYSFSQAKEVFQMYPREINVLHFEEVFK-NK 347
Ot_PPR-TGM      STSSEEEVSAVREHLYSALKDTMGMSDSERKVKRAIRKVISEDGTIRFDRMFSHRLKKDE 481

Jd_TGM          GRLVVEIGSGQGECVVHAAQQASDT-DFLALEVYVPGIASTLYRIRRTGVSNVRVIHADA 153
Mt_TGM          ARLVVEIGSGQGHQIVSAASADPDT-DFLAVEVFTAGLARTMLDADREGVKNLRLVEANA 144
Ag_TGM          APLTIEIGSGTGEQLVAAAAAHPDR-DYLALEVWVPGIAKLLSKAAGAGVENIRVLEADA 157
Ns_TGM          KEVWIEIGFGSGRHLLYQAQQHPDT-IFIGIEIHKPSVEQVLKQIALKDLKNLYIIDYDA 182
Sk_TGM          REVRIEVGFGSARHLLHQAAANPDV-LFIGLEIHKPSIEQALKQIVIQNITNIMIIDYDA 180
Dd_PPR-TGM      RSLKMEICSGHGHWVTERAGQDLDA-DWISLEIRYDRIFQIWSKMILEAIDNLYIVGGDA 626
Bp_PPR-TGM      MKAKLEVCSGHGDWITSRCAKEKETTEWFGIEMRENRVALTWIKSLRLGVRNLTMLCGLA 494
Eh_PPR-TGM      NPINIELCSGYGEWLITKAEEKKDE-NWVGVELYRDRVYNSWATKVFAGLDNVACVWGDA 406
Ot_PPR-TGM      RELNLEVAAGNGDWAVAQAATDDSS-DWISLELRHDRVYSIFSRAVFSGASNFAAMGGDA 540
                    :*:  * .      .    .   :..:*:    :             *.  :   *

Jd_TGM          AQAVDTYLPAAGVDEVWIFFPDPWHKSRHHKRRLIQPAFLDRLTRVLRPGGVVRLATDWQ 213
Mt_TGM          PEVLEHMLPAASVDELWVFFPDPWHKNKHTKRRLVAPEFARIAAQALRPGGTLRLATDWQ 204
Ag_TGM          AQALPHLLGEATAREVWTFFPDPWRKARHRKRRLVSDAFALEVARLLEDGGAWRMATDWD 217
Ns_TGM          R-LFLEFVPSNVVGKIFVHFPVPWDKKPH--RRVFSKPFIEEAKRVLKVDGVLELRTDSI 239
Sk_TGM          R-LFLEFVPSNIVGKIYVHFPVPWDKKPH--RRVISEAFLKESERALRVGGTLELRTDSE 237
Dd_PPR-TGM      HGSLKEIIPDNILNEVYINYPNPP--VWSGAERLINELFLIEINRCLKKDGTLTIVTDDK 684
Bp_PPR-TGM      HECMRKQIPNEVLDEIYVNFPDPPEWNGS-ANCLVDGAFLVESHRTLKTGAYLILVTDDP 553
Eh_PPR-TGM      MNVLMLNIMADSIDNIYLNFPEPPKYENS-PTNLFTIQFFFQVARILKLDGVFCLLTDSP 465
Ot_PPR-TGM      AYVMRRYIAPGSVSNVFVNFPEPPHHSGDAAADNSLALLNKEKALVIFSDNHRYMQSWLE 600
                   .   :      :::  :* *               :       :  .    : :
```

```
Jd_TGM           DYADHMRETFDAHPQFTLVST------------------DRFEGRPLTSFERKGLEKDRV 255
Mt_TGM           DYADQMRDVLDEAPGFERAFAGE--------------WADRFEGRVLTAFERKGLRVGRD 250
Ag_TGM           DYAWQMRDVVEACPLLENPHAGERPDP-ADPRPDRGGFAPRYEGRIITHFETRGLDAGRR 276
Ns_TGM           NYFEYALDLLLQEKKVKFEVA------------------KNIEPPVSSKYEDRWTRLGKD 281
Sk_TGM           NYYRFALETFSSPAISRFDVR------------------KNQEIAIVSKYEDRWKKMEKN 279
Dd_PPR-TGM       DYSDQIIDILTKSKKLLKIYKPVKET----------YLIQLSEDYGYSYFNKLWNNGQRV 734
Bp_PPR-TGM       GYAMRMCRELSVVPHLFEPTATDGKP----------FENRLPADYGFSYFNALWTNGNLN 603
Eh_PPR-TGM       IVVNIVNKQFAHSEILRNKFQIQGKT---------MFEKKLPEGYGTSYFDQLWKNGKIN 516
Ot_PPR-TGM       CSWYGLFSSRDADVTSREPAELYETIDGVRLYQGIPGPLAGHRVHEQSYFDRFWENGRHV 660
                                                                : ::

Jd_TGM           VTDLAYTVR--------------------------------------------------- 264
Mt_TGM           IRDLTYTRVSP------------------------------------------------- 261
Ag_TGM           AHDIVGVRVPRA------------------------------------------------ 288
Ns_TGM           IYDIRMFNLEFSEQPILHCDFSFETVSYDESKLEQLHFTPQVYDNFFVHFEKLYAIENGG 341
Sk_TGM           IYDVTFTCEAESPEPDIIGNFTFSGGAVDPKIIDRLSTETYKREWGFLHVERTFTVGENG 339
Dd_PPR-TGM       KRYCIYISKIIN------------------------------------------------ 746
Bp_PPR-TGM       DRYYIKYQKRNI------------------------------------------------ 615
Eh_PPR-TGM       QDRFFMKIKKIM------------------------------------------------ 528
Ot_PPR-TGM       DRYFLSVGKVA------------------------------------------------- 671

Jd_TGM           ---------------------------------------------------
Mt_TGM           ---------------------------------------------------
Ag_TGM           ---------------------------------------------------
Ns_TGM           KVIRLSFGSFDRPEHKYLVFKDGNISYFPKKPVLSKANVAAHKKILELLHG 392
Sk_TGM           SMIRLAMGSFDRPESTYVLIENGAARYYPSFPVRSSANLQAHILLDELIHG 390
Dd_PPR-TGM       ---------------------------------------------------
Bp_PPR-TGM       ---------------------------------------------------
Eh_PPR-TGM       ---------------------------------------------------
Ot_PPR-TGM       ---------------------------------------------------
```

Additional file 1: Amino acid sequence alignment of PPR-TGM proteins with bacterial TGMs. Bacterial TGM sequences used in the alignment include those from *Jonesia denitrificans* [Jd, accession no. YP_003160858], *Microbacterium testaceum* [Mt, accession no. YP_004223305], *Actinomyces georgiae* [Ag, accession no. ZP_16358116], *Nitratiruptor* sp. [Ns, YP_001356223] and *Sulfuricurvum kujiense* [Sk, YP_004060596]. PPR-TGM sequences used in the alignment include those from *Dictyostelium discoideum* [Dd, accession no. XP_646896], *Bathycoccus prasinos* [Bp, accession no. CCO16496], *Entamoeba histolytica* [Eh, accession no. XP_001913841] and *Ostreococcus tauri* [Ot, accession no. XP_003079103]. Identical (*), conserved (:) and semi-conserved (.) amino acids are indicated. The PPR-containing region of the PPR-TGM proteins is denoted by the red box and the TGM domain of all sequences is denoted by the green box.
