# Supplementary material for: Identification of a novel pentatricopeptide repeat subfamily with a C-terminal domain of bacterial origin acquired via ancient horizontal gene transfer
Source: BMC Res Notes. 2013 Dec 9;6:525. doi: 10.1186/1756-0500-6-525 (PMC4029402; doi:10.1186/1756-0500-6-525)
Supplement: Additional file 2 — Amino acid sequence alignment of PPR-TGM proteins with chlamydial TGMs. Chlamydial TGM sequences used in the alignment include those from Parachlamydia acanthamoebae [Pa, accession no. ZP_06300024], Candidatus Protochlamydia amoebophila [Cpa, accession no. YP_008284], Waddlia chondrophila [Wc, accession no. YP_003709095], Simkania negevensis [Sn, YP_004671640], Chlamydophila pneumoniae [Cp, YP_005662431] and Chlamydia psittaci [Cps, accession no. AFS24598]. PPR-TGM sequences used in the alignment include those from Dictyostelium discoideum [Dd, accession no. XP_646896], Bathycoccus prasinos [Bp, accession no. CCO16496], Entamoeba histolytica [Eh, accession no. XP_001913841] and Ostreococcus tauri [Ot, accession no. XP_003079103]. Identical (*), conserved (:) and semi-conserved (.) amino acids are indicated. The PPR-containing region of the PPR-TGM proteins is denoted by the red box and the TGM domain of all sequences is denoted by the green box. [file 1756-0500-6-525-S2.pdf]

```
Pa_TGM          ------------------------------------------------------------
Cpa_TGM         ------------------------------------------------------------
Wc_TGM          ------------------------------------------------------------
Sn_TGM          ------------------------------------------------------------
Cp_TGM          ------------------------------------------------------------
Cps_TGM         ------------------------------------------------------------
Dd_PPR-TGM      MDLKRKFGNSLGSTAPTSIGILGGGGGGVSITKLIVKNIPKSISKNEIIENLKKIIQNPS 60
Bp_PPR-TGM      ------------------------------------------------------------
Eh_PPR-TGM      ------------------------------------------------------------
Ot_PPR-TGM      ------------------------------------------------------------

Pa_TGM          ------------------------------------------------------------
Cpa_TGM         ------------------------------------------------------------
Wc_TGM          ------------------------------------------------------------
Sn_TGM          ------------------------------------------------------------
Cp_TGM          ------------------------------------------------------------
Cps_TGM         ------------------------------------------------------------
Dd_PPR-TGM      INITIPITLPPDLIGKVDADTTISFLSSQNLSQLSIIKNLVNGKTIGDKKVIVDFYDFKK 120
Bp_PPR-TGM      ------------------------------------------------------------
Eh_PPR-TGM      ------------------------------------------------------------
Ot_PPR-TGM      ----------------------------------------------------MAKRKKRP 8

Pa_TGM          ------------------------------------------------------------
Cpa_TGM         ------------------------------------------------------------
Wc_TGM          ------------------------------------------------------------
Sn_TGM          ------------------------------------------------------------
Cp_TGM          ------------------------------------------------------------
Cps_TGM         ------------------------------------------------------------
Dd_PPR-TGM      VIPTPTPIPTPTPPTKTQEESNKKIKLTNEKPKEKKPKEKKPKTTPPPTTTTPNEPTIAT 180
Bp_PPR-TGM      ------------------------MAAKRKNEESREVSDTRENYKKKSSNGVVVGGDFAR 36
Eh_PPR-TGM      -----------------------------------------------MSKESTTPNEINE 13
Ot_PPR-TGM      WLDASVVRNARARDGEKARERRERERAFREKCPERFVEEEKERESERERDARARDGDFKA 68

Pa_TGM          ------------------------------------------------------------
Cpa_TGM         ------------------------------------------------------------
Wc_TGM          ------------------------------------------------------------
Sn_TGM          ------------------------------------------------------------
Cp_TGM          ------------------------------------------------------------
Cps_TGM         ------------------------------------------------------------
Dd_PPR-TGM      ATATTTTTETTETEKTTEINEEKKVNQKKNKKLNDKKEEKEKEKEVKKEEEPKKESIVRN 240
Bp_PPR-TGM      FVAEQQQKDTSKEEHLSSLNELQPTPYSQLAKLDEGMNRCFRANRKRSRDAQTLLKRAKK 96
Eh_PPR-TGM      RKIEEKPHKEEKKMKKQRYKKEEILGNPLEEYFEKVSKNVAPYNPIGRAEEEIEEG---F 70
Ot_PPR-TGM      STSGRGGRRNDREGADGRRKGHKGDGSPDDSFSTDP--RTFPTHATFQTREGLSNDALEW 126

Pa_TGM          ------------------------------------------------------------
Cpa_TGM         ------------------------------------------------------------
Wc_TGM          ------------------------------------------------------------
Sn_TGM          ------------------------------------------------------------
Cp_TGM          ------------------------------------------------------------
Cps_TGM         ------------------------------------------------------------
Dd_PPR-TGM      NRAVINKITSQMSSCAEAKDYVQLVKAYNYLKKIGAKPDHITYGVMLNACVRCQEYKKVK 300
Bp_PPR-TGM      LKEVKFFNRLIRDFGNDKQFGFAEEAFRILEKECNIEPNAYSYTNLLNACVRVGELKRAR 156
Eh_PPR-TGM      SQKSIFYINKLFADAAQERNYGKVIYLYKKLIESGIKEDLHTITNIINSAARVGDGILVD 130
Ot_PPR-TGM      RRSRVEAVTKDIAMCARHKQLRRACRAFVQLIEDGMVPSSYTYASLLNAYVNTGSMDG-A 185

Pa_TGM          ------------------------------------------------------------
Cpa_TGM         ------------------------------------------------------------
Wc_TGM          ------------------------------------------------------------
Sn_TGM          ------------------------------------------------------------
Cp_TGM          ------------------------------------------------------------
Cps_TGM         ------------------------------------------------------------
Dd_PPR-TGM      EVFQDAVKDGNAN--EVVYTIYVKALCEIDMDESFATIKGMIET------KPNIRTFNSI 352
Bp_PPR-TGM      EVFQKMERDCDEKPNEVTCTVFIKGLCEEGLIDEALELVKDMVRGSASRPRANVRTFSTI 216
Eh_PPR-TGM      KTWIR-MKQLGLKANEVTRTVSVKGYFAAGLVEKAMYTYYCMD------NRNNIRSINAA 183
Ot_PPR-TGM      EALMERMSEVGCAPNVVAYTTMLKGYMLVADVDAAWRLLEGMKHP----VAPDIRAVNTY 241
```

```
Pa_TGM          ------------------------------------------------------------
Cpa_TGM         ------------------------------------------------------------
Wc_TGM          ------------------------------------------------------------
Sn_TGM          ------------------------------------------------------------
Cp_TGM          ------------------------------------------------------------
Cps_TGM         ------------------------------------------------------------
Dd_PPR-TGM      FRGCIRSGDIEITKSLIQLM-KANEIYPDSTTIEYLIKIYSH---HLMVQEIWDLLGKVY 408
Bp_PPR-TGM      LRNCVRYRDVHSAEATFSLMRECFDVLPDAACYEYLSKSYAS---RLDVEKAEKTLNELE 273
Eh_PPR-TGM      IRGLLRLGNSKQ----IHTFTKHPINAEDMTTKEYLTALYSI---EQEISLVRKIMESIN 236
Ot_PPR-TGM      IRVCVRCGSLTXXXXXXXSVKKNEHLRWTLDPSFGHCQFWCAGKCERGANCRFYHDPSIE 301

Pa_TGM          ------------------------------------------------------------
Cpa_TGM         ------------------------------------------------------------
Wc_TGM          ------------------------------------------------------------
Sn_TGM          ------------------------------------------------------------
Cp_TGM          ------------------------------------------------------------
Cps_TGM         ------------------------------------------------------------
Dd_PPR-TGM      ERMQGQISPICFSRLSLASLLAGDIKSSVKALGITDDILSKAPRSTQTTHKNKGKLSEKN 468
Bp_PPR-TGM      LQQDENGKVLNIPASALASLAG--VAATVGKVDVAKRAIAKCRERADEEQRNAEQFSSNA 331
Eh_PPR-TGM      NEDEMSATSLIN------------------------------------------------ 248
Ot_PPR-TGM      QVDASARETDVNDMLAHLYVNHAHATAMSGDVKQCFKSLAKAAESFAQDDDGNAAGLKDR 361

Pa_TGM          ------------------------------------------------MKPEDLKSPFRE 12
Cpa_TGM         ------------------------------------------------MKPKDLKSPFKW 12
Wc_TGM          ------------------------------------------------MKPKDLKFPFSW 12
Sn_TGM          ------------------------------------------------MKPKDLKSPFSF 12
Cp_TGM          ------------------------------------------------MKPQDLSPPFLW 12
Cps_TGM         ------------------------------------------------MKPQDLKAPFFW 12
Dd_PPR-TGM      KISSSLFERINKQEINEESDRVRNYLSKMTESRSKIKYHNMESFNRIYFSKNTFKNNVNR 528
Bp_PPR-TGM      NSSSEKDRSNNQKPNDDSTTPSKSVSNFFKARASDALREIVEIEAFLSSDDDVIRQEAKQ 391
Eh_PPR-TGM      --LATLNCLIGDVETGDLLLKEFEDISKRETDARKSIRLFKKHQVAQLRNRRKHIRTFIL 306
Ot_PPR-TGM      DERAELFRQTSRDELKREMKRIKAFAERLKNGEQKAPNLDEHFARSLIFSSQILQPPERG 421
                                                                 .

Pa_TGM          GERKV---------------------LIQDRVWYIPLKGLSESDPFRFPGWEDPALFGNS 51
Cpa_TGM         EHRQI---------------------VIQDRVWYVPDQHLSD-IPFIFPGWTHDQTFAQD 50
Wc_TGM          EARQV---------------------LLKDRVLYVP-EYYDNYDQFTFPGWEAPSLFGNS 50
Sn_TGM          RDRRP---------------------CILERIFYVP-TYYDKYKEFSFPFWQE--LFGND 48
Cp_TGM          KERRP---------------------CIQDGVLYVP-RHYFEHQNFSTSYHQE--FFQNH 48
Cps_TGM         EERKT---------------------QIKDDVLYIP-EHYFKHNQFEMPSWEE--FFGNN 48
Dd_PPR-TGM      SIDQQ---------------------CKPVTVATSPDSTLSSFNGLFNSYKKGGSNFNSN 567
Bp_PPR-TGM      RAEAFG-------------------VDETDDIVFVHKQRELAETPAIEKFWASRYERKGK 432
Eh_PPR-TGM      NTQPIL-------------------KEQRKKYSFSQAKEVFQMYPREINVLHFEEVFKNK 347
Ot_PPR-TGM      STSSEEEVSAVREHLYSALKDTMGMSDSERKVKRAIRKVISEDGTIRFDRMFSHRLKKDE 481

Pa_TGM          S--PVYVEYCSGNGTWIAEKAAENP-SINWVAVEKKFERVRKIWSKIKNLQLPNLIVISG 108
Cpa_TGM         K--PIYLEYCSGNGAWIASKAIEQS-QYNWVGIEKKFDRTRKIWSKIKKFELDNLLTICG 107
Wc_TGM          K--SVVIEYCSGNGAWIVEKAKSNP-QNNWVAVEKRFDRVRKIWSKIKNEHLDNLVVFCG 107
Sn_TGM          H--PVHIEYCSGNGEWILERAKAHP-EVNWVAVEMKFERVRKIHSKRINRNISNLLIVCG 105
Cp_TGM          T--SIACELCSGNGDWVVAQAQKDP-QVLWIAVEQRFDRVRKIWSKMINHQIQNLRIVCG 105
Cps_TGM         H--PIFCELCSGNGDWVVAQANKNP-NMNWIAVEKRFDRVRKIWSKMHNSQVRNLRIVCG 105
Dd_PPR-TGM      R--SLKMEICSGHGHWVTERAGQDL-DADWISLEIRYDRIFQIWSKMILEAIDNLYIVGG 624
Bp_PPR-TGM      AFMKAKLEVCSGHGDWITSRCAKEKETTEWFGIEMRENRVALTWIKSLRLGVRNLTMLCG 492
Eh_PPR-TGM      N--PINIELCSGYGEWLITKAEEKK-DENWVGVELYRDRVYNSWATKVFAGLDNVACVWG 404
Ot_PPR-TGM      R--ELNLEVAAGNGDWAVAQAATDD-SSDWISLELRHDRVYSIFSRAVFSGASNFAAMGG 538
                       * .:* * *   :.  .     *..:*   :*              *.  . *
```

```
Pa_TGM          EGLLTTERFFPQESVEAVYVNFPDPWPKKRHAKNRIIQPIFVQQIARILKPSGS------ 162
Cpa_TGM         EGFHVTRDFFANESVDAVFINFPDPWPKKRHAKHRIVQPAFVKEIHRILKSDKL------ 161
Wc_TGM          EGFRMTQEYFPSGAVDRIYINFPDPWPKKRHAKHRIVQRPFVEQISRILKPQGV------ 161
Sn_TGM          EAQVFTREYVPENSVETIYVNFPDPWPKDRHAKHRLIQAPFMRDVLTAVRPGGN------ 159
Cp_TGM          TAETFFQYYVPDQFLQRLVVNFPDPWPKMRHRKHRLLQPSFVQEISRSLQDSAV------ 159
Cps_TGM         EAQTFFRHYIQNEVIQRIVVNFPDPWPKSRHRKHRLFQDEFMNDIVRVLVDSGI------ 159
Dd_PPR-TGM      DAHGSLKEIIPDNILNEVYINYPN--PPVWSGAERLINELFLIEINRCLKKDGT------ 676
Bp_PPR-TGM      LAHECMRKQIPNEVLDEIYVNFPDP-PEWNGSANCLVDGAFLVESHRTLKTGAY------ 545
Eh_PPR-TGM      DAMNVLMLNIMADSIDNIYLNFPEP-PKYENSPTNLFTIQFFFQVARILKLDGV------ 457
Ot_PPR-TGM      DAAYVMRRYIAPGSVSNVFVNFPEPPHHSGDAAADNSLALLNKEKALVIFSDNHRYMQSW 598
                 .       .    :. : :*:*:                :  :    :

Pa_TGM          ------LTLVTDSLEYSAQMIEVVNGSVDFSSYFP----APYYSEDVQNYGSSYFEQLWK 212
Cpa_TGM         ------FTLVTDDAAYSKIMIDVIYQTGGFISLFE----APYYTTSYPGYGSSYFEELWR 211
Wc_TGM          ------LTLVTDDAVYSEQMIEVLNMQEGMCSAHP----EPYYVNELEGYGTSFFDALWR 211
Sn_TGM          ------AQLVSDAKEYVLQMQAEISQVSGWK---------PSNHQNQNNYGSSYFDRLWR 204
Cp_TGM          ------FALATDDKTYLLESIEALQT--RLAPRME----TPYYIKMTDTYGNSWFENLWR 207
Cps_TGM         ------IILATDDKNYLLQAIKIMQQ--RLLPQLE----EPYYCKMLENYGDSWFERLWR 207
Dd_PPR-TGM      ------LTIVTDDKDYSDQIIDILTKSKKLLKIYKPV-KETYLIQLSEDYGYSYFNKLWN 729
Bp_PPR-TGM      ------LILVTDDPGYAMRMCRELSVVPHLFEPTATD-GKPFENRLPADYGFSYFNALWT 598
Eh_PPR-TGM      ------FCLLTDSPIVVNIVNKQFAHSEILRNKFQIQGKTMFEKKLPEGYGTSYFDQLWK 511
Ot_PPR-TGM      LECSWYGLFSSRDADVTSREPAELYETIDGVRLYQG---IPGPLAGHRVHEQSYFDRFWE 655
                        : :            .                         :  *:*: :*

Pa_TGM          EQGRIIRYHQFQKKIMEEACSF 234
Cpa_TGM         EKGKSIHYHVFRKI-------- 225
Wc_TGM          DKGRVIRYHQYRKQELMHAVD- 232
Sn_TGM          SLGREIYYLEYLCEK------- 219
Cp_TGM          TKGQEIFYTEFIKKAGI----- 224
Cps_TGM         SKGQEIFYTEFVKKVGI----- 224
Dd_PPR-TGM      NGQRVKRYCIYISKIIN----- 746
Bp_PPR-TGM      NGNLNDRYYIKYQKRNI----- 615
Eh_PPR-TGM      NGKINQDRFFMKIKKIM----- 528
Ot_PPR-TGM      NGRHVDRYFLSVGKVA------ 671
```

Additional file 2: Amino acid sequence alignment of PPR-TGM proteins with chlamydial TGMs. Chlamydial TGM sequences used in the alignment include those from *Parachlamydia acanthamoebae* [Pa, accession no. ZP_06300024], *Candidatus Protochlamydia amoebophila* [Cpa, accession no. YP_008284], *Waddlia chondrophila* [Wc, accession no. YP_003709095], *Simkania negevensis* [Sn, YP_004671640], *Chlamydophila pneumoniae* [Cp, YP_005662431] and *Chlamydia psittaci* [Cps, accession no. AFS24598]. PPR-TGM sequences used in the alignment include those from *Dictyostelium discoideum* [Dd, accession no. XP_646896], *Bathycoccus prasinos* [Bp, accession no. CCO16496], *Entamoeba histolytica* [Eh, accession no. XP_001913841] and *Ostreococcus tauri* [Ot, accession no. XP_003079103]. Identical (*), conserved (:) and semi-conserved (.) amino acids are indicated. The PPR-containing region of the PPR-TGM proteins is denoted by the red box and the TGM domain of all sequences is denoted by the green box.
