# Supplementary material for: Identification of a novel pentatricopeptide repeat subfamily with a C-terminal domain of bacterial origin acquired via ancient horizontal gene transfer
Source: BMC Res Notes. 2013 Dec 9;6:525. doi: 10.1186/1756-0500-6-525 (PMC4029402; doi:10.1186/1756-0500-6-525)
Supplement: Additional file 3 — Phylogenetic tree displaying the relationship of PPR-TGM proteins to chlamydial TGMs using phylogenetic software program, Phylogeny.fr. Amino acid sequences were aligned using MUSCLE. Bacterial rRNA methyltransferases were used as the outgroup. The maximum likelihood phylogeny tree was generated using the Jones-Taylor-Thornton model. The scale represents the number of substitutions per site. Statistical support for the branches was ascertained via bootstrapping (100 replicates). [file 1756-0500-6-525-S3.pdf]

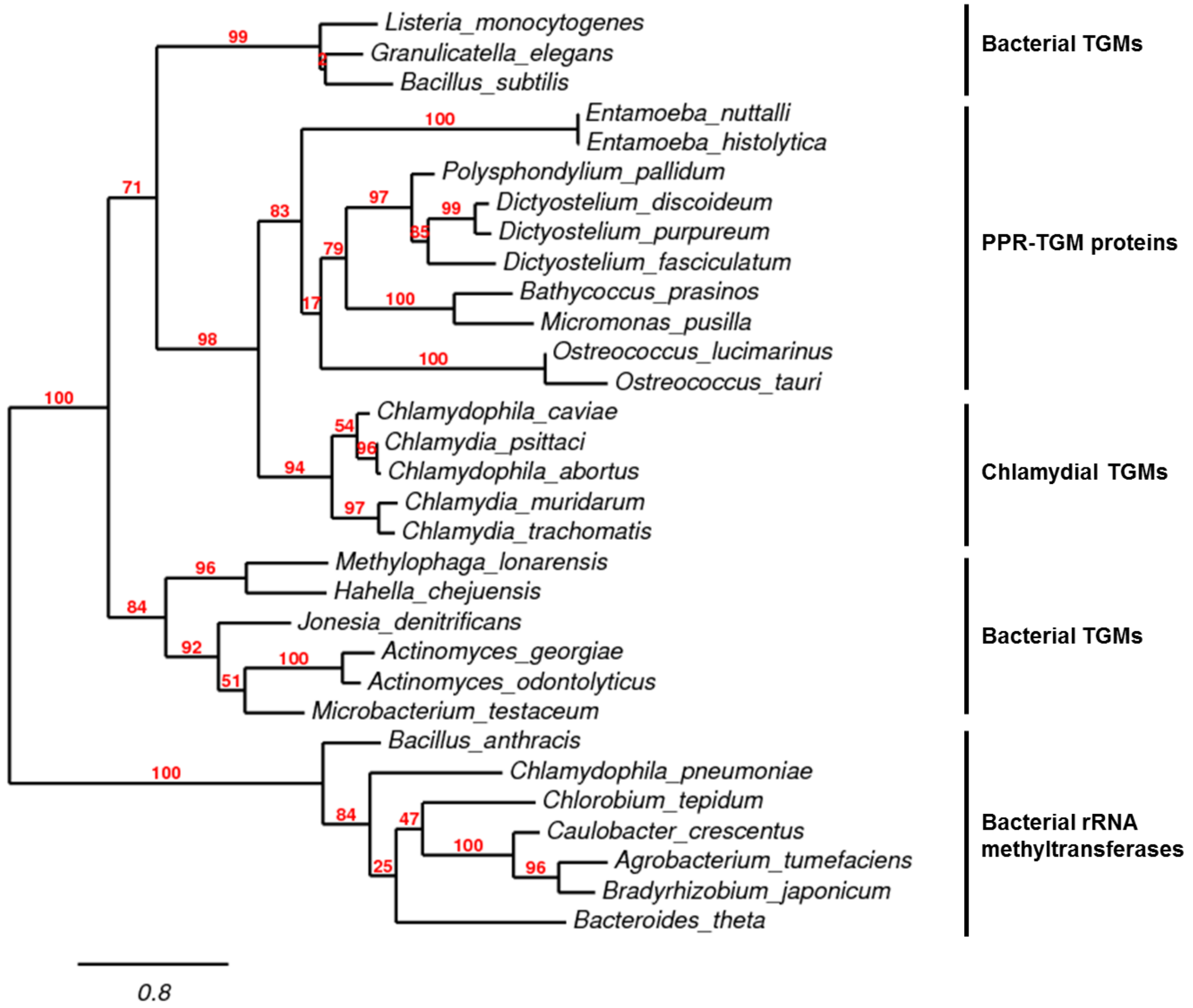


**Additional file 3: Phylogenetic tree displaying the relationship of PPR-TGM proteins to chlamydial TGMs using phylogenetic software program, Phylogeny.fr.** Amino acid sequences were aligned using MUSCLE. Bacterial rRNA methyltransferases were used as the outgroup. The maximum likelihood phylogeny tree was generated using the Jones-Taylor-Thornton model. The scale represents the number of substitutions per site. Statistical support for the branches was ascertained via bootstrapping (100 replicates).
